# Spécificités de la réorganisation de la prise en charge des patients cancéreux au cours de la pandémie COVID-19 dans un centre régional d´oncologie au Maroc

**DOI:** 10.11604/pamj.supp.2020.35.23280

**Published:** 2020-07-15

**Authors:** Ghizlane Rais, Bouchra Amaoui, Laila Lahlou, Mohammed Ouazni, Salma Fares

**Affiliations:** 1 Medical Oncology Department, Regional Center of Oncology, Agadir, Morocco,; 2 Radiotherapy Department Regional Center of Oncology, Agadir, Morocco,; 3 Public Health Department, Faculty of Medicine and Pharmacy Agadir, University IBN Zohr Agadir,; 4 Surgery Department, Faculty of Medicine and Pharmacy Agadir, University IBN Zohr Agadir,; 5 Hematology Department, Faculty of Medicine and Pharmacy Agadir, University IBN Zohr Agadir

**Keywords:** COVID 19, pandemie, soins contre le cancer, Agadir, Maroc

## Résumé

Les patients atteints de cancer présentent un risque élevé de développer une forme grave, voire mortelle, d’infection SARs-CoV-2. Dans cette pandémie de crise sanitaire, cette population de patients doit bénéficier d’un traitement rapide qui doit être adapté au contexte épidémiologique. Au Maroc et notamment au Centre Régional d’Oncologie d’Agadir, un groupe de travail local s’est réuni pour proposer des conduites de prise en charge des patients cancéreux dans ces circonstances inhabituelles. La situation épidémiologique actuelle au Maroc et précisément dans la région sud est toujours sous contrôle et la gestion des patients cancéreux doit prendre en compte le risque de perte de chances de guérison pour ces patients. Nous suggérons que les possibilités de continuité des soins soient sauvegardées avec une adaptation des différents traitements au contexte épidémique et prérogatives gouvernementales.

## Essay

Le Coronavirus disease 2019 (COVID-19) constitue actuellement un problème de santé à l´échelle mondiale avec de lourdes répercussions sanitaires, sécuritaires, économiques et sociales [1]. L´agent causal est le SARS-COV-2, une souche à fort potentiel de contagion qui a fait son apparition à Wuhan en chine, au mois de décembre 2019 [2]. Depuis l´annonce de la pandémie COVID-19 en mars 2020, et afin d´essayer de protéger un système sanitaire déjà fragile, le Maroc a adopté très tôt des mesures drastiques d´hygiène et de sécurité collective et individuelle à grande échelle, notamment en optant pour le confinement général et le port obligatoire de masque [3]. Plus particulièrement, l´impact que pouvait avoir cette pandémie sur les patients cancéreux était une préoccupation majeure des professionnels de la santé. Un rapport actualisé de l’OMS démontre une mortalité de 7,6% chez les patients atteints de cancer [4]. En effet, ces patients sont considérés comme un groupe très vulnérable par rapport à la population générale en raison de leur état immunosuppresseur dû à la malignité, à la chimiothérapie et aux comorbidités. Ainsi, les oncologues sont obligés de reconsidérer les traitements anticancéreux en tenant compte du risque de complication et de progression du cancer [5]. Par ailleurs, la situation des nouveaux cas parait encore plus complexe compte tenu des risques de retard diagnostique et de prise en charge qui pourrait faire perdre aux patients la chance de guérison.

En réponse à la propagation du COVID-19, et compte tenu de la particularité du centre régional d´oncologie d´ AGADIR au Maroc sur le plan géographique et l´ évolutivité de l´infection COVID-19 au sud du Maroc encore maitrisé, un groupe de travail regroupant oncologue médical, onco-hématologue, radiothérapeute, chirurgien et épidémiologiste du centre régional d´oncologie s´est réuni afin d´établir des recommandations sur l´organisation globale et spécifique de la prise en charge des patients cancéreux en cette période de crise. Ce centre anti-cancéreux, même s´il se trouve au sein d´un hôpital prenant en charge la plupart des cas d´infection COVID de la région est pour le moment épargné de la prise en charge du COVID-19 mais se prépare à une éventuelle saturation des formations sanitaires avoisinantes.

**Données internationales et nationales sur le COVID-19 chez le patient cancéreux:** le nouveau bilan de l’évolution de la pandémie du COVID-19 dans le monde a provoqué, à ce jour, 221 823 décès dans 214 pays dénombrés depuis son apparition, avec 3 145 407 cas confirmés, dont plus de 1 000 000 guéris [1]. Les données disponibles à ce jour sur la base des expériences chinoises et italiennes récentes, suggèrent que le taux d´infection au COVID-19 serait 2 à 3 fois plus important chez les sujets atteints de cancer par rapport à la population générale, Par ailleurs, les patients cancéreux présentent un risque de complications respiratoires sévères imposant une prise en charge en réanimation et de décès plus élevé [5-7]. L’âge avancé est le facteur de risque majeur associé aux événements graves dus à l’infection par le SRAS-CoV-2 chez les patients atteints de cancer. Les patients ayant reçu une chimiothérapie ou une chirurgie dans les deux semaines qui précédent l’infection avait également un risque plus important de développer des complications respiratoires sévères (OR = 5,34, p = 0,0026). Dans une étude italienne, 20% des décès dus au COVID-19 dans tout le pays étaient chez des patients atteints d’un cancer actif [7]. Ces données sont néanmoins hétérogènes et d´autres études sont nécessaires.

**Figure 1:**
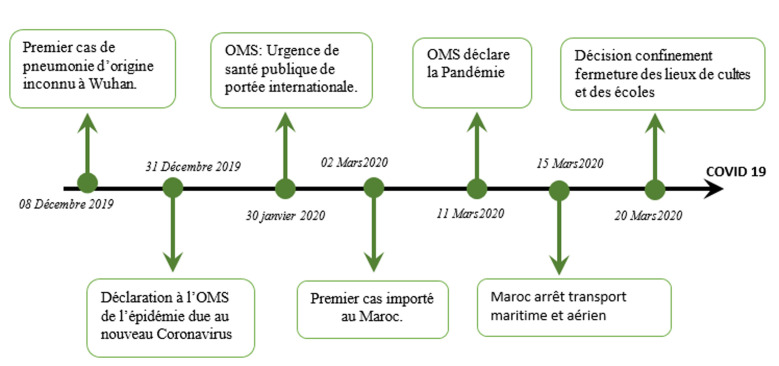
chronologie du plan de riposte au Maroc

En ce qui concerne le Maroc, le début de l´épidémie était lié à la déclaration du premier cas le 02 mars chez un patient revenant d´Italie [8]. La plupart des cas enregistrés par la suite était des cas importés, jusqu´au 30 mars où la découverte de « clusters » locaux annonce l´entrée en phase 2 de l´épidémie, avec plus de 80% des cas issus de contamination locale. La proportion des cas enregistrés dans les 4 régions du Sud du Maroc que couvre le centre régional d´oncologie représente 1.9%(89 cas) des 4523 cas de COVID-19 enregistré au Maroc jusqu´au 01 mai 2020. En effet, la situation épidémiologique au sud connait une accélération de la vitesse d´apparition des cas depuis le 18 avril 2020, après une période de stabilité, l´effectif des cas cumulés est de 89 cas confirmés avec l´enregistrement de clusters familiaux. Le Maroc a évité jusqu´à présent une catastrophe sanitaire grâce à l´application large de mesures drastiques incluant la fermeture des frontières terrestres, aériennes et maritimes depuis le 15 mars 2020, l´arrêt des études pour tous les niveaux scolaires et universitaires à partir du 16 mars 2020. Un confinement strict de la population depuis le 20 mars, une hygiène préventive stricte (lavage des mains, utilisation d´antiseptiques et de désinfectants, les gestes barrières), et le port obligatoire du masque par la population dans tous les lieux publiques (Figure 1) ont été également adoptés.

Parallèlement à ces mesures générales, le ministère de la santé a adopté très tôt un plan de riposte basé sur une adaptation évolutive dans le temps de l´ensemble du secteur de la santé à la situation épidémiologique liée au COVID-19. Un redéploiement des structures de réanimations et des personnels de la santé vers la prise en charge des patients infectés, si l´évolution de la pandémie l´impose est adopté. Les directives du ministère ont aussi insisté sur une sanctuarisation tant que possible des centres d´oncologies afin d´épargner une population atteinte de cancer vulnérable et souvent immunodéprimée. Par ailleurs, un plan de renforcement de l´accessibilité du traitement pour les malades cancéreux éloignées des centres d´oncologie pour assurer leur transport a été mis en place afin d´assurer la continuité des soins pour ces patients [9].

**Figure 2:**
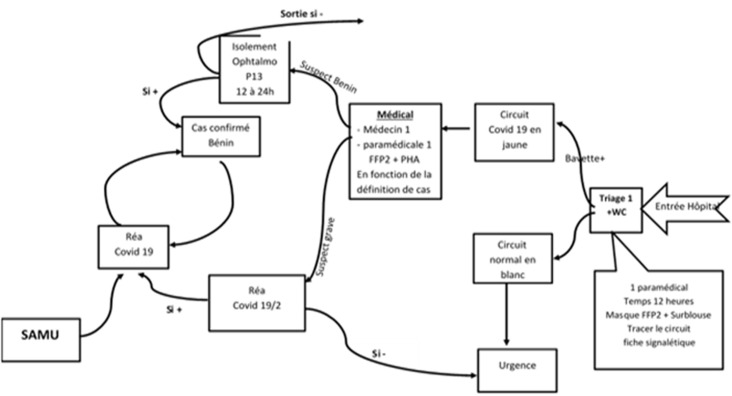
modèle opérationnel du circuit des malades du Centre Hospitalier Régional d´Agadir dans le contexte de l´épidémie du COVID-19

**Les mesures d´organisation générale pour la prise en charge des patients atteints de cancer:** au Maroc, comme la plupart des pays dans le monde, une réorganisation des soins dans les centres anticancéreux pendant la pandémie de COVID-19 était cruciale et plusieurs sociétés savantes ont récemment proposé des recommandations essentielles, aussi bien pour les patients recevant actuellement des traitements actifs que ceux en suivi, ainsi que pour l’admission des patients et leurs soignants à l’hôpital [10-15]. Depuis le début de cette crise sanitaire inédite, dans notre institution, un groupe de travail multidisciplinaire s´est engagé activement dans l´élaboration d´un plan d´action visant d´une part le soutien et la sécurité du personnel soignant et des patients et d´autre part la continuité des soins. Ce groupe a veillé aussi sur l´élaboration de propositions concernant la hiérarchisation des soins et l´organisation de la prise en charge des patients. Une nouvelle réorganisation de l´accès des patients et leurs accompagnants au centre d´oncologie a été mise en place. A l´entrée de la porte réservée aux patients, une unité de tri a été installée. Un interrogatoire sur les symptômes de COVID-19 ainsi que la prise de température sont réalisés pour le patient. Pour les cas suspects le patient est référé à l´unité médicale spécialisée COVID-19 pour approfondir le diagnostic (cette unité se trouvant à l´hôpital régional situé à proximité du centre d´oncologie où il doit bénéficier d´une TDM thoracique systématique en plus du test de dépistage de COVID). Si un cas est déclaré positif alors le traitement pour le patient en question sera différé jusqu´à sa rémission complète de l´infection. Les patients qui n´ont pas de symptômes de l´infection COVID-19 peuvent accéder au centre munis d´une bavette chirurgicale pour réaliser leur traitement (Figure 2).

L´accès au centre a été limité à un seul accompagnant si besoin (perte d´autonomie, fin de vie) et une seule visite par jour d´une heure. Cette nouvelle réorganisation a permis également d´aménager les salles d´attente en plein air du centre d´oncologie, de telle sorte à laisser une distance acceptable entre les patients. Des affiches desservies partout dans le centre ont été mis au point afin d´inciter les patients et leurs familles à appliquer les gestes barrières, incluant le port de masque, et les mesures d´hygiène et à respecter le confinement à domicile en dehors des jours où ils se présentent à l´hôpital. D´autres mesures importantes ont été adoptées afin d´assurer la sécurité des patients, leurs accompagnants éventuellement et les personnels soignants. Ces mesures consistent à: a) espacer et optimiser le temps de la consultation; b) éviter l´attente des patients; c) reporter les consultations à caractère non urgent en téléphonant aux patients; d) privilégier la télémédecine; e) ne pas accepter d´accompagnant sauf si sa présence est indispensable; f) mettre à l´entrée du centre de consultation des solutions hydro alcooliques pour la désinfection des mains; g) port de masque chirurgical obligatoire pour tous les patients; h) réduire le nombre de patients au niveau de la salle d´attente avec au moins 2 mètres entre les patients; i) la salle de consultation doit être aérée, ensoleillée et doit disposer d´un point d´eau avec du savon liquide avec distributeur et/ou solution hydro alcoolique; j) le port indispensable de masque FFP2 pour le médecin et le personnel soignant et administrateur de l´admission; k) désinfecter après chaque patient: table d´examen, stéthoscope; l) mettre en place des poubelles pour déchets d’activités de soins à risques infectieux DASRI.

### L´organisation spécifiques aux différentes spécialités

**Organisation de l´activité du service d´oncologie médicale au cours de la pandémie COVID-19:** en se référant aux récentes recommandations émises par les sociétés savantes internationales [10-16] et nationales, notre groupe de travail a adapté ces recommandations au contexte épidémiologique local où les structures de soins ne sont pas encore saturées. Parallèlement aux mesures générales mises en place, nous avons émis des propositions pour adapter le traitement anti-cancéreux en fonction du stade de la maladie, du bénéfice du traitement et de l´état général en prenant en considération le contexte social du patient et son éloignement du lieu de traitement et la problématique de l´organisation de leur transport a été également l´un des paramètres à prendre en compte. On peut distinguer ainsi trois situations cliniques.

Patients en cours de traitement: ces patients constituaient l´activité principale du centre. Les principales recommandations sont de: a) évaluer le bénéfice risque pour chaque patient; b) ne pas retarder l´initiation d´un traitement curatif; c) les patients atteints de cancers localisés ou considérés comme curables doivent être traités dans les meilleurs délais; d) poursuivre des traitements adjuvants et néo-adjuvants à visée curative et à haute valeur ajoutée; e) des approches locorégionales non chirurgicales doivent être discutées en RCP devant une atteinte pauci-métastatique; f) imiter les réunions physiques de concertation multidisciplinaire RCP et privilégier les RCP virtuelles; g) privilégier les schémas administrés toutes les trois semaines plutôt que les protocoles hebdomadaires; h) poursuivre toutes les hormonothérapies et les thérapies ciblées orales (exemple sunitinib dans le cancer du rein métastatiques); i) les traitements per os (thérapeutiques ciblées, hormonothérapie, chimiothérapie) sont à privilégier pendant cette période s´ils donnent des résultats équivalents aux chimiothérapies cytotoxiques habituelles administrées par voie intraveineuse; j) la majorité des protocoles contenant le cisplatine devraient être switchés à la carboplatine, à l´exception de certaines situations où il y a un bénéfice majeur telles que les tumeurs germinales; h) utilisation des facteurs de croissance hématopoïétique pour les chimiothérapies à risque intermédiaire de neutropénie afin de réduire le risque d´aplasie fébrile; i) imiter les bilans au minimum afin d´éviter le risque d´expositions au COVID-19; j) discuter cas par cas les malades en situation palliative présentant des stades avancés; k) réduire tant que possible l´usage des corticoïdes à visée anti-hémétique et les anti-inflammatoires à visée antalgique; l) pour les chimiothérapies palliatives: la discussion de chaque patient tiendra compte du bénéfice attendu, de l´âge et des comorbidités. Nous avons suggéré de poursuivre la chimiothérapie chez les patients présentant un état général conservé et un bénéfice du traitement en cours, en particulier pour les premières lignes de traitement. L´indication sera réduite probablement selon le stade de l´épidémie. Nous avons maintenu les chimiothérapies en association avec les thérapies ciblées (exemple maintenir une mono ou bichimiothérapie en association avec bevacizumab dans les cancers colorectaux métastatiques; poursuivre le trastuzumab en association à la chimiothérapie toutes les 3 semaines en première ligne dans le cancer du sein métastatique); j) espacer les bilans d´évaluation et agir selon le bénéfice clinique.

Concernant l´unité d´hospitalisation, le centre dispose d´un seul service commun entre les trois spécialités (oncologie médicale, radiothérapie et hématologie) avec une capacité litière limitée. En situation normale, cette unité est dédiée aux patients recevant des protocoles de chimiothérapie longs ou nécessitant des précautions particulières (exemple hyperhydratation pour cisplatine) ou nécessitant des soins palliatifs. En situation de pandémie, la gestion de l´unité d´hospitalisation consistait à privilé gier l´hospitalisation des urgences oncologiques et onco-hématologique: patients présentant des complications dues soit à la maladie cancéreuse (compression médullaire, hypertension intra-cranienne.) soit à la toxicité des traitements reçus (thrombopénies sévères, neutropénie fébrile).

**Patients en cours de surveillance:** ces patients seront appelées par téléphone pour reporter leur rendez-vous de consultations après vérification de l´absence de signes cliniques de rechute ou progression. Si un bilan radiologique est demandé, celui-ci doit être également reporté jusqu´au nouvel rendez-vous accordé. Leur ordonnance peut être renouvelée.

**Nouveaux cas:** tous les nouveaux cas avec un diagnostic histologique ont été pris en charge. Un bilan minimaliste a été préconisé selon la situation clinique. Nous avons privilégié plutôt la tomodensitométrie. Nous avons choisi d´initier le traitement selon l´urgence de la situation oncologique, le caractère curatif ou palliatif de la maladie et selon le bénéfice potentiel des traitements préconisés.

**Organisation de l´activité de l´onco-hématologie au cours de la pandémie COVID-19:** les patients suivis pour une hémopathie représentent une population particulièrement fragile, du fait de l´immunodépression secondaire à leur affection hématologique, et/ou de certaines thérapeutiques indiquées. Dans un institut d´hématologie à Wuhan en Chine, l´observation des patients atteints d´hémopathies graves, en particulier d´hémopathies malignes, ont conclu que les patients infectés par le SARS-CoV-2 au cours de la chimiothérapie présentaient des symptômes atypiques et plus de complications. Le SARS-CoV-2 a montré une transmission extrêmement rapide et répandue parmi les patients du service d’hématologie, avec une mortalité très élevée (> 50%) pour les patients hospitalisés atteints à la fois du SARS-CoV-2 et une hémopathie maligne [17]. En parallèle avec les mesures générales déjà citées et qui rejoignent les autres spécialités dans leur organisation, des mesures particulières en fonction de la pathologie peuvent être adoptées afin d´optimiser la prise en charge des patients suivis en hématologie. L´objectif principal est de maintenir une stratégie thérapeutique adaptée et optimale sans retentissement majeur sur le pronostic, tout en protégeant les patients et le personnel soignant d´une surexposition au COVID-19. Plus que de véritables recommandations, des pistes de réflexions, des propositions ou des priorisations ont été proposées par les sociétés savantes internationales ou nationales pour la prise en charge des patients d´hématologie dans le contexte de la propagation de l´épidémie COVID-19 [18-21]. Dans notre contexte, nous avons essayé de maintenir une activité de consultation et d´hospitalisation subnormale tout en évitant au maximum d´admettre un patient COVID-19.

Concernant les hémopathies malignes aiguës comme les leucémies aiguës, la mise en route d’une prise en charge urgente après avoir éliminé une éventuelle infection par le SAR-COV 2 restera une priorité. Les traitements d’induction vont être maintenus tout en discutant la possibilité de rajouter du G-CSF afin de raccourcir la durée de la neutropénie. Au cours des consolidations, une réduction des doses d’Aracytine pour les leucémies myéloïdes aiguës ou une éventuelle suppression de corticoïdes pour les leucémies aiguës lymphoïdes pourraient être discutées. Si un patient COVID-19 est admis, la chimiothérapie doit être arrêtée ou décalée jusqu´à résolution de l´infection tout en gardant le patient sous un traitement symptomatique. Le traitement par chimiothérapie curative des lymphomes non hodgkiniens (LNH) agressifs et du lymphome de Hodgkin (LH) doit être maintenu tout en évitant les protocoles intensifs ou les procédures d´intensification avec support des cellules souches autologues, utiliser le G-CSF pour raccourcir la durée de neutropénie, et supprimer les traitements de maintenance par Rituximab. La radiothérapie curative dans le LH devra être maintenue dans les stades localisés. Concernant les hémopathies lymphoïdes indolentes, il s´agit plutôt de reporter l´initiation du traitement si c´est possible sinon en cas d´urgence thérapeutique, éviter au maximum les traitements induisant une lymphodéplétion et prioriser les traitements per os.

Pour le myélome multiple symptomatique, le traitement per os doit être privilégié, en réduisant les doses de dexamethasone voire une suppression si le patient répond, sinon opter pour le bortézomib hébdomadaire, différer l´autogreffe des cellules souches, totaliser 6 à 8 cures d´induction puis entretenir par le Lenalidomide ou le Thalidomide, et envisager d´étendre l´accès aux immuno-modulateurs jusqu´à 2 mois aux patients recevant un traitement de maintenance (tests télémédecine, laboratoires à distance entre les deux). En ce qui concerne, la leucémie myéloïde chronique, le traitement par les inhibiteurs de tyrosine kinase devra être maintenu sans changement en dehors d´un espacement des visites et les évaluations de la réponse moléculaire ou cytogénétique qui seront faites à 3- 6 mois de traitement. Les traitements de support y compris les transfusions peuvent aussi faire l´objet d´adaptation vu la situation actuelle, en proposant un abaissement des seuils transfusionnels des produits sanguins labiles: HB < 7 g/dl ou si syndrome anémique mal toléré et pour les plaquettes < 10 Giga/L avec un syndrome hémorragique. Sinon, maintenir les perfusions d´immunoglobulines si indication vu la non disponibilité de la forme sous cutanée au Maroc.

**Organisation de l´activité de radiothérapie au cours de la pandémie COVID-19:** le groupe de travail a proposé une organisation transitoire de l´activité du service de Radiothérapie durant cette période pandémique. Des circuits fiables pour le personnel ainsi que pour les patients sont installés et les protocoles de traitement sont adaptés à la situation COVID-19 actuelle. Plusieurs scénarios seront envisageables pour faire face à toute situation pandémique ultérieure. Selon le risque de contamination probable nous avons subdivisé le service en trois zones: a) une zone propre représente le circuit ou le risque de contamination est faible (les bureaux administratifs la salle de dosimétrie et la salle de repos du personnel); b) une zone semi-contaminée englobe le circuit ou la probabilité de contamination est moyen (les vestiaires, les couloirs des patients, les toilettes ainsi que les salles d’attentes); c) une zone contaminée présente le circuit ou le risque de contamination est important (la salle de simulation, le pupitre de l’accélérateur et la salle de traitement). Nous avons aussi veillé sur la désinfection des différentes zones par l´instauration d´une procédure par zone. Dans la zone propre, toutes les surfaces doivent être nettoyées quotidiennement avec de l´éthanol à 75%. Une désinfection terminale en fin de consultation et en fin de traitement doit être appliquée pour la zone semi-contaminé. Pour la zone contaminée une désinfection deux fois par jour doit être appliquée [22].

Pour accéder au service, nous avons exigé qu´il se fasse par deux issus. Une réservée strictement aux personnels et l´autre aux patients. A l´entrée de la porte réservée au personnel, chaque membre de l´équipe doit déclarer à la commission de suivi du personnel s´il présente des symptômes suspects et sa température va être prise. Le personnel soignant bénéficie de masques chirurgicaux (changés toute les 4h à 6h), d´une visière ainsi que d´une solution hydro alcoolique. Le déplacement du personnel entre les unités se fera dans un circuit interne sans aucun contact avec les patients. Un système de roulement par activité a été choisi (simulation, contouring, consultation de surveillance et contrôle qualité au poste de traitement). Par ailleurs, la radiothérapie est un traitement «salvateur» qui doit être garantie à tous les patients cancéreux pour lesquels est indiqué [23]. Par contre, un ajustement des protocoles de traitement pendant la pandémie doit être préconisé. La stratégie que nous avons adoptée privilégie les protocoles hypo-fractionnés car permet de réduire la charge de travail et d´exposer le patient à moins de risques d´infection [10]. Les patients traités à titre curatif avec des tumeurs en place (col utérin, ORL et poumon) ont été priorisé. Pour les cas palliatifs en absence d´une autre alternative thérapeutique nous avons adopté une irradiation mono-fractionnée ou au maximum 5 fractions. La consultation de surveillance hebdomadaire n´est systématique que pour les patients en cours de chimiothérapie concomitante. Pour les patients symptomatiques, ils seront vus au cours de leur séance de radiothérapie. Pour les consultations post-thérapeutiques, nous avons privilégiés la téléconsultation pour éviter ainsi le déplacement des patients tout en maintenant le contact patients-médecins. La gestion des ressources humaines en cas d´aggravation de l´état épidémique dans la région.

**L´activité chirurgicale et le contexte pandémique:** l´activité chirurgicale oncologique a été la plus affectée par cette crise sanitaire. En effet, afin de s´adapter à l´évolution épidémiologie rapide, la directive globale du ministère de la santé imposait une mobilisation de vive allure mettant en jeu tous les moyens disponibles pour contenir ce fléau. Parmi les mesures essentielles concernant l´activité chirurgicale, on cite: a) la réduction de la capacité litière d´hospitalisation; b) le désengagement des services de réanimations pris par la prise en charge des patients COVID-19; c) le redéploiement du personnel (réanimateurs, anesthésistes, infirmiers du bloc opératoire) vers la structure de prise en charge des patients COVID-19.

Le principe général de la réorganisation de l´activité chirurgicale était de déprogrammer toute activité? chirurgicale non urgente, et sans préjudice de perte de chance pour les patients [5,6,24-26] tout en essayant de: a) limiter les situations à risque très élevé: association chirurgie majeure et poly-chimiothérapie cytotoxique; b) limiter les contacts et en particulier avec les lieux de soins. Par ailleurs, Les chirurgies carcinologiques induisent une immunodépression modérée. Le sur-risque de morbi-mortalité post-opératoire induit par la pandémie doit être mis en balance avec la potentielle perte de chance d´un retard de prise en charge chirurgicale oncologique. Afin d´adapter l´activité de la chirurgie oncologique dans ce contexte particulier plusieurs sociétés savantes ont émis des propositions plutôt que des recommandations afin d´orienter la prise en charge chirurgicale [27]: a) ne pas arrêter toute activité de chirurgie oncologique mais plutôt l´adapter au contexte épidémique; b) le maintien des chirurgies avec un faible taux de complications et ne nécessitant pas un séjour en réanimation tels que les traitements chirurgicaux des urgences oncologiques, du cancer du sein, du cancer colique. c) discuter préférentiellement l´indication de traitement néoadjuvant des patients éligibles permettant soit de retarder la chirurgie soit de l´éviter (exemple: dans le cancer du sein, discuter la possibilité d´hormonothérapie néoadjuvante si cancer du sein avec récepteurs hormonaux positifs ou de chimiothérapie néoadjuvante chez les patients ayant un cancer du sein triple négatifs ou HER2 positif même en cas de petite tumeur); d) en cas de comorbidités, de projet de chirurgie complexe ou longue, il est important d´envisager la pertinence de l´indication opératoire dans le contexte COVID-19 avec les anesthésistes-réanimateurs de l´équipe de réanimation qui serait potentiellement sollicitée.

Concernant le Bilan préopératoire, il est conseillé de vérifier le statut COVID du patient peu avant la chirurgie si celui-ci présente des signesévocateurs de cette infection ou en cas d´intervention portant sur lesvoies aériennes ou ses annexes (Haut risque de contamination carelles favorisent l´aérosolisation du virus). Certaines précautions sontindispensables en salle d´intervention, notamment il est recommandéde: a) restreindre au minimum le nombre de soignants autour despatients suspects, possibles ou confirmés; b) procéder au nettoyage dessurfaces du mobilier du bloc opératoire à l´aide de l´eau de Javel prêteà l´emploi ou à diluer ou à l´aide de produits détergents/désinfectantsvirucides (norme NF EN 14476). Il est important de noter que la datede fin de l´épidémie n´est pas connue et une fois celle-ci atteinte lepersonnel médical et paramédical sera amoindri (personnels infectés et/ou épuisés) avec pour conséquence des délais de « reprogrammation »pouvant être longs.

## Conclusion

Les patients atteints de cancer sont confrontés à une double peine, le COVID-19 et le cancer. Les cancérologues doivent aussi faire face à un contexte inédit. Les incertitudes sur la durée de l´épidémie ne doivent pas faire repousser l´instauration d´un traitement curatif du cancer et ajouter ainsi une mortalité par cancer à la mortalité due au COVID-19. Il est nécessaire également de maintenir le contact des patients cancéreux avec le personnel médical, paramédical et les structures sanitaires pour ne pas ajouter un sentiment d´abandon au stress du confinement dans un parcours de soins déjà complexe et anxiogène.

### Etat des connaissances sur le sujet

- La pandémie de COVID-19 est un défi mondial sans précédent qui nécessite un effort mondial pour la contrôler et gérer;
- Des recommandations ont été mises en place pour la protection des patients cancéreux souvent fragiles et immunodéprimés;
- La réorganisation et la hiérarchisation des soins contre le cancer durant la pandémie du COVID-19 est indispensable.

### Contribution de notre étude à la connaissance

- Décrire un modèle de réorganisation des soins contre le cancer dans un centre régional d´oncologie au Maroc malgré des moyens limités;
- Des mesures générales et spécifiques adaptées au contexte épidémiologique permettraient de sauvegarder les chances de guérison aux patients cancéreux.

## Remerciements

Nous remercions tout le personnel médical, infirmier et administratif du centre régional d´oncologie pour les efforts fournis durant cette pandémie.
